# Field evaluation of the efficacy of *Mycobacterium bovis* BCG vaccine against tuberculosis in goats

**DOI:** 10.1186/s12917-017-1182-5

**Published:** 2017-08-17

**Authors:** Enric Vidal, Claudia Arrieta-Villegas, Miriam Grasa, Irene Mercader, Mariano Domingo, Bernat Pérez de Val

**Affiliations:** 1 IRTA, Centre de Recerca en Sanitat Animal (CReSA, IRTA-UAB), Campus de la Universitat Autònoma de Barcelona, Bellaterra, 08193 Barcelona, Catalonia Spain; 2 Departament de Sanitat i Anatomia Animals, Universitat Autònoma de Barcelona, Bellaterra, 08193 Barcelona, Catalonia Spain; 3 Associació de Ramaders de Cabrum de Catalunya, 25400 Les Borges Blanques, Lleida, Catalonia Spain; 4 Departament d’Agricultura, Ramaderia, Pesca i Alimentació de la Generalitat de Catalunya, 08007 Barcelona, Catalonia Spain

**Keywords:** BCG, Efficacy, Field study, Goats, Tuberculosis, Vaccine

## Abstract

**Background:**

Control of animal tuberculosis (TB) through vaccination has emerged as a long-term strategy to complement test and slaughter control strategy. A pilot trial under field conditions was conducted in a goat herd with high TB prevalence to assess the efficacy of the *Mycobacterium bovis* BCG vaccine.

**Results:**

Twenty-three goat kids vaccinated with BCG and other 22 unvaccinated control kids were euthanized at 18 months post-vaccination. Gross pathological and histopathological examination of target tissues was performed for detection of tuberculous lesions and assessment of vaccine efficacy. Mycobacterial culture and DNA detection were used to confirm *Mycobacterium caprae* infection. Vaccination significantly reduced the number of animals with TB lesions compared to unvaccinated controls (35% and 77%, respectively; *P* < 0.01). This reduction was even higher if only extra-pulmonary infection was considered (17% and 68%, respectively; *P* < 0.001).

**Conclusions:**

This trial demonstrates that BCG vaccination of goats can significantly reduce the TB lesion rates in high disease exposure conditions, indicating that vaccination could contribute to the control of TB in domestic goats.

## Background

Animal tuberculosis (TB) is a chronic infectious disease caused by mycobacteria of the *Mycobacterium tuberculosis* complex (MTBC), mainly *M. bovis* and *M. caprae*, which affects a wide range of domestic and wild animals. TB in goats causes economic losses in endemic areas due to trade limitations, and depopulation of positive herds, when test and slaughter strategy fails [1, 2]. Furthermore, infected goats have been identified as a source of bovine TB in mixed goats-cattle herds or nearby cattle farms in non-officially bovine TB-free EU countries [3, 4]. Unlike TB in cattle, no official EU policy exists for TB control in goats, thus hindering the success of bovine TB eradication programs.

Vaccination may be a control option to reduce disease prevalence in animals in high-prevalent herds where the test and slaughter strategy could be inefficient, expensive in terms of cost-benefit, logistically demanding or could threaten valuable genetic resources [5, 6]. Also, vaccination could be an affordable tool to control TB in underdeveloped countries unable to afford conventional test and slaughter protocols [7–9].

*M. bovis* bacillus Calmette-Guérin (BCG) is an appealing vaccine for use in livestock and wildlife as its use is licensed for humans, has an excellent safety record and is relatively inexpensive to manufacture [10]. The efficacy of BCG in the field has been assessed in cattle [7, 11] and in wildlife TB hosts, such as possums or badgers [12, 13]. Furthermore, our research group has also recently demonstrated that BCG vaccination of goats affords a degree of protection against experimental challenge with *M. caprae* [14, 15].

Even though vaccination of cattle with mycobacterial vaccines is at present prohibited in EU counties, the implementation of vaccination campaigns in goats is not expressly prohibited and would not require national and EU laws and directives. Here we report the first trial to assess the efficacy of BCG vaccination of goat kids under field conditions.

## Methods

### Herd and experimental schedule

A TB positive herd of Murciana-Grandina goats, located in Catalonia, with confirmed *Mycobacterium caprae* (spoligotype profile SB0416) infection and a reactor rate of 79% to the single intradermal comparative cervical tuberculin (SICCT) test, was selected for this study. A lot of 45 new born, approximately 1 month old, goat kids (16 males and 29 females) from the herd reposition were included in the study. Animals were randomly divided in two groups of 23 (8 males and 15 females) and 22 (8 males and 14 females), respectively. This sample size was deemed appropriate to estimate differences of proportions between treatment groups, i.e. 25% and 66% estimated proportion of infection in vaccinated and unvaccinated groups respectively (95% confidence interval, 90% statistical power, one-tailed test; source: www.winepi.net, University of Zaragoza).

At day 0, all experimental animals were negative to the SICCT test and the IFN-γ release assay (Bovigam™, Thermo Fisher Scientific, Schlieren, Switzerland). Twenty three animals were vaccinated subcutaneously with approximately 10^5^ colony forming units (CFU) *M. bovis* BCG Danish strain (ATCC, Ref. 35,733™), prepared as previously described [16], and the other 22 remained as unvaccinated controls. Two months after vaccination, the kids were mixed with the rest of the herd and remained exposed to natural infection for 16 months, after this period, all the animals included in the study were humanely sacrificed.

All animals were fed following the regular farm conditions throughout the study (i.e. colostrum/milk initially, followed by forage supplemented with feed). Animals were followed-up for clinical signs every two months approximately.

### Necropsy, tissue sampling and histopathology

The 45 experimental animals were euthanized by intravenous sodium pentobarbital overdose and carefully post-mortem examined in order to detect tuberculous lesions. Euthanasia was performed at 18 months after BCG vaccination. A complete necropsy procedure was conducted as previously described [17]. Briefly, tracheobronchial and mediastinal lymph nodes (LN) were aseptically removed making sure that the pleural lung surface was not sectioned, were sliced and examined for the presence of TB-like gross lesions, and then were stored at −20 °C as a pulmonary LN pool. Whole lungs were fixed with 10%-buffered formalin by pouring the fixative into the trachea. One month later, lungs were sliced at 1 cm thick intervals for TB-like gross lesion examination. All remaining viscera were also examined and all TB-like gross lesions detected in other tissues were collected at necropsy and subsequently fixed with 10%-buffered formalin, embedded in paraffin and examined by histopathology (haematoxylin and eosin and Ziehl Neelsen staining). For each animal at least one section of any tissues with visible lesions was included in the paraffin block, samples of the mesenteric lymph node and ileum were always included regardless whether lesions were observed or not macroscopically. The whole slide was evaluated under the microscope using the different magnification available in the microscope (4×, 10×, 20 x and 40× and, in the case of the Ziehl Neelsen Staining, the 100× magnification with immersion oil was used). The slides were analysed blindly by a pathologist. TB indicative lesions were set when the following histopathological features were observed: Presence of granulomas, with or without central necrosis and mineralization, surrounded by macrophages, multinucleated giant cells, sometimes partially or completely encapsulated. Enumeration (when present) of acid fast bacilli (AFB) was also performed. Four different categories were defined: None detected (no AFB detected after evaluation of the whole lesion under 100× magnification); 1 (observation of one AFB in at least one 100× field); 2–5 (observation of 2 to 5 AFB in at least one 100× field), >5 (observation of more than 5 AFB in at least one 100×).

### MTBC culture

For microbiological culture, the pulmonary LN pools were thawed and a quantitative mycobacterial culture was subsequently carried out following the procedure described in previous studies [17]. Briefly, LN pools were homogenized, decontaminated, suspended and 100 μl of this suspension were twice serial diluted 1:10. Afterwards, 100 μl of each suspension dilution (1:100, 1:1000 and 1:10,000) were inoculated onto 7H11 agar plates (BD Diagnostics, MD, USA). The inoculated media were incubated at 37 °C. Bacterial counts were performed after 28 days and total CFUs in each pool of pulmonary LN were calculated. Isolates were confirmed as MTBC by multiplex PCR [18]. Then, DNA samples from MTBC isolates were analysed by DVR-spoligotyping [19].

### Direct DNA detection

Once TB lesions were confirmed in non-respiratory tissues by histopathology, DNA extraction was carried out as following: Excess paraffin was trimmed off the sample block using a scalpel, and then 10 μm thick sections were obtained and dewaxed with xylene and absolute ethanol. Once the ethanol supernatant was removed and the remaining ethanol had evaporated the tissue was weighed to obtain an amount of 25 mg. Afterwards, DNA from resulting tissue powder was extracted using a DNA purification kit (Promega Biotech Iberica, Madrid, Spain). A semi-nested PCR, targeted to the MTBC-specific IS*6110* sequence [17] was run under standard conditions and a *M. avium* subsp. *paratuberculosis* (*MAP*)-specific Real Time PCR (Vacunek, Derio, Spain) was run following the manufacturer instructions.

### Serology

Blood samples were collected from all experimental animals at the end point of the study. Sera from all animals were analysed by ELISA to detect antibodies against MTBC as previously described [17] and against *MAP* (ID Screen® Paratuberculosis indirect, ID.vet, Grabels, France).

### Data analysis

Differences in presence of TB lesions and histopathological parameters between groups were assessed by chi-square test. Significance was set at *P* value <0.05. Statistical analysis was performed with R package v2.15.0 (R Foundation for Statistical Computing, Vienna, Austria).

## Results

Besides occasional coughing, experimental animals did not show remarkable clinical signs throughout the experiment. No adverse reactions at BCG injection site were observed at necropsy.

A total of 25 out of 45 goats showed gross and histopathological TB lesions. The proportion of animals with TB lesions was significantly higher in unvaccinated (77%, 95% CI: 54–91) than in vaccinated goats (35%, 95% CI: 17–57; *P* < 0.01). There were no significant differences between sex categories (Table 1).

**Table 1:** Distribution of TB cases by experimental groups, sex and lesion location

		No. of goats with TB lesions (%)^a^			
Group	Sex	Only pulmonary lesions^b^	Only extra-pulmonary lesions^c^	Both pulmonary and extra-pulmonary lesions	Total
BCG	Male (*N* = 8)	1 (13)	0 (0)	2 (25)	3 (38)
(*N* = 23)	Female (*N* = 15)	3 (20)	2 (13)	0 (0)	5 (33)
	Total	4 (17)	2 (9)	2 (9)	8 (35)
Control	Male (*N* = 8)	0 (0)	5 (63)**	1 (13)	6 (75)
(*N* = 22)	Female (*N* = 14)	2 (14)	8 (57)*	1 (7)	11 (79)
	Total	2 (9)	13 (59)***	2 (9)	17 (77)**

^a^TB lesions were determined by histopathology

^b^Lungs and mediastinal and tracheobronchial lymph nodes

^c^Ileum and mesenteric and retropharyngeal lymph nodes

*P < 0.05, **P < 0.01, ***P < 0.001; Chi-square test

**Fig. 1:**
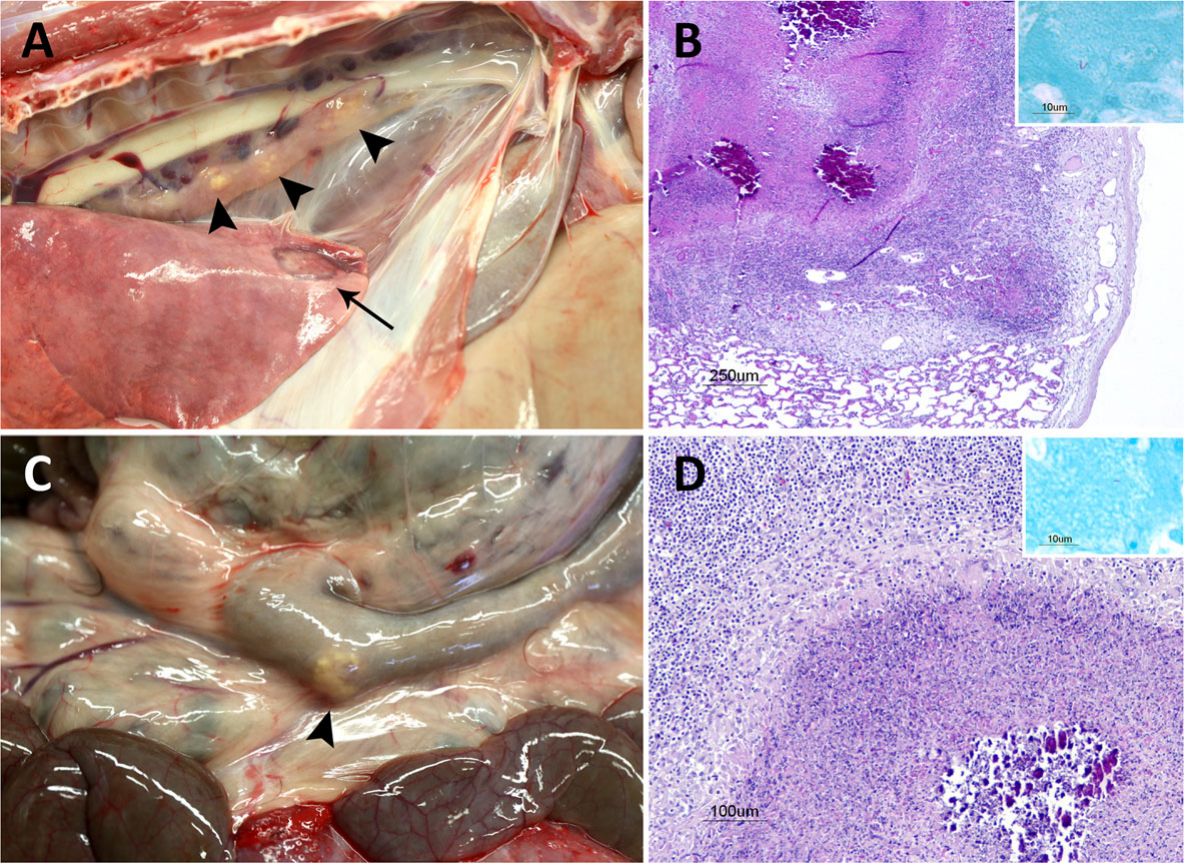
Study of tuberculous lesions. **a** Goat showing a pulmonary tuberculous granuloma (*arrow*) and tuberculous lesions in the mediastinal lymph node (*arrowheads*). **b** Histopathological study of the lung lesion observed in **a**. The insert shows Ziehl-Neelsen stained mycobacteria. **c** Goat with tuberculous granulomatous lymphadenitis lesions in the mesenteric lymph node (*arrowhead*). **d** Histopathology of the lesions observed in C, showing paubacillary tuberculous granulomatous lymphadenitis. The insert shows a Ziehl-Neelsen positive bacillus

Ten goats, 6 vaccinated and 4 unvaccinated, showed pulmonary lesions (Fig. 1a-b, Table 1). MTBC were isolated from pulmonary LN (10^2^ CFU/pooled LN) in 2 of them (both vaccinated). In both cases the strain isolated was identified as *M. caprae* with the spoligotype profile SB0415 (www.Mbovis.org). Nineteen goats showed extra-pulmonary TB lesions confirmed by histopathology (Fig. 1c-d, Table 1). The proportion of goats with extra-pulmonary lesions was significantly higher in unvaccinated than in vaccinated goats (68% and 17%, respectively; *P* < 0.001). All these animals (*N* = 19) showed TB lesions in mesenteric LN. The proportion of AFB positive results in mesenteric LN was significantly lower in BCG vaccinated compared to control animals (25% and 80%, respectively; *P* < 0.05). BCG vaccinated animals also showed a higher (yet not statistically significant) proportion of granulomas in mesenteric LN without necrosis, mineralization or fibrotic capsule compared to control animals (50% and 13%, respectively). A characterization of granulomas in mesenteric LN for each group is shown in Table 2. MTBC DNA was detected in one of them, whereas *MAP* DNA was not detected in any sample. In addition, 5 (2 vaccinated and 3 unvaccinated) and 3 (1 vaccinated and 2 unvaccinated) animals showed TB lesions in retropharyngeal LN and ileum wall, respectively (data not shown).

**Table 2:** Histopathological analysis of granulomas in mesenteric lymph nodes

		Group	
Parameter		BCG (*N* = 4)	Control (*N* = 15)
		No. (%)	No. (%)
Capsule	Absent	2 (50)	2 (13)
	Partial	1 (25)	3 (20)
	Complete	1 (25)	10 (67)
Necrosis	Absence	2 (50)	2 (13)
	Presence	2 (50)	13 (87)
Mineralization	None	2 (50)	2 (13)
	Low	0 (0)	9 (60)
	High	2 (50)	4 (27)
No. of AFB	None detected	3 (75)	3 (20)
	1	1 (25)	3 (20)
	2–5	0 (0)	5 (33)
	>5	0 (0)	4 (27)

*AFB* Acid fast bacilli

At the end of the study, 5 out of 22 unvaccinated goats were positive to the MTBC ELISA (four of them with TB visible lesions), whereas none of the unvaccinated goats were positive to the *MAP* ELISA. Within the BCG vaccinated group, 16 out of 23 goats were positive to the MTBC ELISA (only one with TB visible lesions), and one of them (which had no visible TB lesions) was also positive to the *MAP* ELISA.

## Discussion

We report herein, for first time, a pilot vaccination trial under field conditions that has demonstrated a degree of protection of goats vaccinated with BCG against natural exposure to MTBC. Similar field studies conducted in cattle also showed protective efficacy of BCG seen as a reduction of pathological scores and the number of animals positive to bacteriological culture or immunological tests [7, 11].

Our results are also consistent with those obtained in previous laboratory BCG efficacy studies in goats experimentally infected with *M. caprae* [14, 15], that showed reduction of extra-pulmonary TB cases in vaccinated goats. Unfortunately the quantitative culture method carried out, which started by platting a 1/100 dilution of the pool of whole lymph node homogenates, irrespectively of the extension or localization of lesions, and also included a sample freezing and defrosting step, may have hampered the mycobacterial detection by culture. Thus, even though *M. caprae* infection was confirmed in the herd and in 2 experimental animals (with the same spoligotype profile previously identified in the herd), the vaccine efficacy assessment had to rely on histopathological results.

Interestingly, in the present study most unvaccinated animals showed only extra-pulmonary lesions, mainly in mesenteric LN. In all these cases the lesions showed consistent histopathological features that are strongly indicative of TB. MTBC DNA was detected in one of these lesions confirming the ethiology. Even though other diseases, such as paratuberculosis might occasionally induce similar lesions, the possibility that extra-pulmonary lesions were caused by *MAP* was ruled out: No diffuse granulomatous infiltrates (the most frequent lesions in paratuberculosis infection) were observed in any animal, *MAP* DNA was not detected from any extra-pulmonary lesion, and the 13 goats that only showed extra-pulmonary lesions were all negative to *MAP* ELISA, whereas 5 of them were positive to MTBC ELISA at the end point of the study. Thus the possibility that extra-pulmonary lesions were caused by *MAP* was ruled out. The location of TB lesions in unvaccinated animals suggests that the digestive route of infection could be particularly important in the studied farm, probably due to shared feed and water points with the rest of the farmed animals. These results suggest that BCG vaccination might reduce the number of animals infected through the oral route, and non-respiratory bacterial shedding from infected animals, thus leading to a lower risk of horizontal transmission within the herd.

In high caprine TB prevalent regions, where infected goats may act as domestic reservoirs of TB in cattle [3], the ultimate goal should be to eradicate the disease in goats, along with the eradication of TB in cattle. Hence, our results indicate that vaccination of the entire herd, or at least of the rearing kids, could be useful in reducing the intra-herd prevalence. This strategy could be combined with slaughtering of goats positive to ante-mortem DIVA tests until all infected goats are removed from the flock. Accordingly, some authors have suggested that the effect of BCG vaccination in a targeted population of badgers would result in a gradual decrease in the number of infected individuals leading to a reduction in interspecies transmission, and, if the aim of the vaccination was to eradicate TB from badgers, it would need to be continued until the last infected badger was removed from the population [10, 20].

## Conclusion

The results of this study demonstrate that BCG vaccinated goats in field conditions showed not only reduced MTBC infection rates, but also a significant reduction of extrapulmonary TB. These results indicate that vaccination may be a long-term prospect to reduce the prevalence in herds with a high number of infected animals. A vaccination program, in addition to other disease reduction practices, such as good herd management, biosafety practices and an efficient DIVA tests, may contribute to eradicate caprine TB in endemic areas.

## Abbreviations

BCG: Bacillus Calmette-Guérin
DIVA: Differention of Infected and Vaccinated Animals
IFN-γ: Interferon gamma
LN: lymph nodes
MAP: *M. avium* subsp. *paratuberculosis*
MTBC: *Mycobacterium tuberculosis* complex.
SICCT: single intradermal comparative cervical tuberculin
TB: Tuberculosis

## References

[1] Daniel R, Evans H, Rolfe S, de la Rua-Domenech R, Crawshaw T, Higgins RJ, Schock A, Clifton-Hadley R (2009). Outbreak of tuberculosis caused by Mycobacterium Bovis in golden Guernsey goats in great Britain. Vet Rec.

[2] Seva J, Menchén V, Navarro JA, Pallarés FJ, Villar D, Vásquez F, Bernabé A (2002). Caprine tuberculosis eradication program: an immunohistochemical study. Small Rumin Res.

[3] Napp S, Allepuz A, Mercader I, Nofrarías M, López-Soria S, Domingo M, Romero B, Bezos J, Pérez de Val B (2013). Evidence of goats acting as domestic reservoirs of bovine tuberculosis. Vet Rec.

[4] Zanardi G, Boniotti MB, Gaffuri A, Casto B, Zanoni M, Pacciarini ML (2013). Tuberculosis transmission by Mycobacterium Bovis in a mixed cattle and goat herd. Res Vet Sci.

[5] Krebs JR, Group TISR (1997). Bovine tuberculosis in cattle and badgers.

[6] Vordermeier HM, Pérez de Val B, Buddle BM, Villarreal-Ramos B, Jones GJ, Hewinson RG, Domingo M (2014). Vaccination of domestic animals against tuberculosis: review of progress and contributions to the field of the TBSTEP project. Res Vet Sci.

[7] Ameni G, Vordermeier M, Aseffa A, Young DB, Hewinson RG (2010). Field evaluation of the efficacy of Mycobacterium Bovis Bacillus Calmette-Guerin against bovine tuberculosis in neonatal calves in Ethiopia. Clin Vaccine Immunol.

[8] Conlan AJK, Brooks Pollock E, McKinley TJ, Mitchell AP, Jones GJ, Vordermeier M, Wood JLN (2015). Potential benefits of cattle vaccination as a supplementary control for bovine tuberculosis. PLoS Comput Biol.

[9] Murphy D, Corner LAL, Gormley E (2008). Adverse reactions to Mycobacterium Bovis bacille Calmette-Guérin (BCG) vaccination against tuberculosis in humans, veterinary animals and wildlife species. Tuberculosis (Edinb).

[10] Buddle BM, de Lisle GW (2014). The role of vaccination in the control of tuberculosis in badgers. Vet J.

[11] Lopez-Valencia G, Renteria-Evangelista T, Williams J de J, Licea-Navarro A, la M-VAD, Medina-Basulto G (2010). Field evaluation of the protective efficacy of Mycobacterium Bovis BCG vaccine against bovine tuberculosis. Res Vet Sci.

[12] Chambers MA, Rogers F, Delahay RJ, Lesellier S, Ashford R, Dalley D, Gowtage S, Dave D, Palmer S, Brewer J, Crawshaw T, Clifton-Hadley R, Carter S, Cheeseman C, Hanks C, Murray A, Palphramand K, Pietravalle S, Smith GC, Tomlinson A, Walker NJ, Wilson GJ, Corner LA, Rushton SP, Shirley MD, Gettinby G, McDonald RA, Hewinson RG (2011). Bacillus Calmette-Guerin vaccination reduces the severity and progression of tuberculosis in badgers. Proc Biol Sci / R Soc.

[13] Tompkins DM, Ramsey DS, Cross ML, Aldwell FE, de Lisle GW, Buddle BM (2009). Oral vaccination reduces the incidence of tuberculosis in free-living brushtail possums. Proc Biol Sci / R Soc.

[14] Pérez de Val B, Villarreal-Ramos B, Nofrarías M, López-Soria S, Romera N, Singh M, Abad FX, Xing Z, Vordermeier HM, Domingo M (2012). Goats primed with Mycobacterium Bovis BCG and boosted with a recombinant adenovirus expressing Ag85A show enhanced protection against tuberculosis. Clin Vaccine Immunol.

[15] Pérez de Val B, Vidal E, Villarreal-Ramos B, Gilbert SC, Andaluz A, Moll X, Martí-n M, Nofrarías M, McShane H, Vordermeier HM, Domingo M (2013). A multi-antigenic adenoviral-vectored vaccine improves BCG-induced protection of goats against pulmonary tuberculosis infection and prevents disease progression. PLoS One.

[16] Pérez de Val B, Vidal E, López-Soria S, Marco A, Cervera Z, Martín M, Mercader I, Singh M, Raeber A, Domingo M (2016). Assessment of safety and interferon gamma responses of Mycobacterium Bovis BCG vaccine in goat kids and milking goats. Vaccine.

[17] Pérez de Val B, López-Soria S, Nofrarías M, Martín M, Vordermeier HM, Villarreal-Ramos B, Romera N, Escobar M, Solanes D, Cardona PJ, Domingo M (2011). Experimental model of tuberculosis in the domestic goat after Endobronchial infection with Mycobacterium Caprae. Clin Vaccine Immunol.

[18] Wilton S, Cousins D (1992). Detection and identification of multiple mycobacterial pathogens by DNA amplification in a single tube. Genome Res.

[19] Kamerbeek J, Schouls L, Kolk A, van Agterveld M, van Soolingen D, Kuijper S, Bunschoten A, Molhuizen H, Shaw R, Goyal M, van Embden J (1997). Simultaneous detection and strain differentiation of mycobacterium tuberculosis for diagnosis and epidemiology. J Clin Microbiol.

[20] Gormley E, Corner LAL (2011). Control of tuberculosis in badgers by vaccination: where next?. Vet J.

